# Does Drinking Reduce Stress?

**Published:** 1999

**Authors:** Michael A. Sayette

**Affiliations:** Michael A. Sayette, Ph.D., is an associate professor in the Department of Psychology, University of Pittsburgh, Pittsburgh, Pennsylvania

**Keywords:** AOD (alcohol or other drug) use, psychological stress, tension reduction theory, family AODU (AOD use, abuse, and dependence) history, personality trait, cognition, gender differences, context dynamics, temporal context, theoretical model, literature review

## Abstract

For centuries, people have used alcohol to relieve stress—that is, the interpretation of an event as signaling harm, loss, or threat. The organism usually responds to stress with a variety of behavioral, biological, and cognitive changes. Alcohol consumption can result in a stress-response dampening (SRD) effect, which can be assessed using various measures. Numerous individual differences and situational factors help determine the extent to which a person experiences SRD after consuming alcohol. Individual differences include a family history of alcoholism, personality traits, extent of self-consciousness, cognitive functioning, and gender. Situational factors influencing alcohol’s SRD effect include distractions during a stressful situation and the timing of drinking and stress. The attention-allocation model and the appraisal disruption model have been advanced to explain the influence of those situational factors.

Since antiquity, people have observed a complex relationship between alcohol consumption and stress. Not only have stressful situations induced drinking, but alcohol consumption also has long been considered a way of relieving stress. For example, more than 2,500 years ago, the Greek lyric poet Alcaeus suggested drinking as a way to cope with distress: “ We must not let our spirits give way to grief . . . Best of all defenses is to mix plenty of wine, and drink it.” 1 Similarly, Shakespeare referred to alcohol’s stress-reducing properties in his play *Julius Caesar* (Act IV, Scene III): “ Speak no more of her. Give me a bowl of wine. In this I bury all unkindness. . . .” The concept that alcohol can “calm the nerves” is, in fact, widely held across cultures. In the United States, both social drinkers (i.e., people who consume alcohol within socially accepted limits and who experience no alcohol-related problems) and problem drinkers (i.e., people who experience alcohol-related social, medical, or legal problems) believe in alcohol’s stress-reducing properties. The media and the entertainment industry also consistently portray drinking as a way to relieve stress (Wilson 1988). Researchers believe that alcohol’s anticipated stress-relieving effect is a primary motivation for many people to consume alcohol, despite the often harmful consequences of drinking (Sayette 1993*a*).

Clinicians and researchers also have noted the relationship between alcohol consumption and stress. In the 1940s, sociological investigations suggested a link between the level of stress in certain non-Western cultures and the rates of problem drinking (see Pohorecky 1991). Around the same time, Masserman conducted experiments demonstrating that alcohol administration could reduce conflict-induced stress in cats (Masserman and Yum 1946; also see Sayette 1993*a*). Subsequently, Conger’s (1956) theory regarding alcohol’s reinforcing properties led to the development of the tension-reduction hypothesis. The hypothesis comprises two separate propositions: (1) under most circumstances, alcohol consumption will reduce stress, and (2) in times of stress, people (or animals) will be especially motivated to drink alcohol.

This article reviews human studies investigating the first part of the tension-reduction hypothesis—namely, whether drinking reduces stress. (The second part of the hypothesis—i.e., stress induces alcohol consumption—is discussed in other articles within this journal issue.) The current article first defines and provides information on the assessment of stress. It then summarizes various individual and situational factors that may influence susceptibility to alcohol-induced stress reduction and describes evidence supporting the role of those factors.

## Definition and Assessment of Stress

Historically, the term “stress” has been used to describe both the stimuli or events (i.e., stressors) that disturb an organism and the organism’s complex physiological response to such a stimulus (i.e., the stress response). Because people respond to the same stimulus in different ways, however, Lazarus and Folkman (1984) suggested that stress may best be defined as the appraisal or interpretation of an event as signaling harm, loss, or threat. This approach, which this article also adopts, recognizes that an event may be construed as stressful by one person but interpreted as harmless or positive by another person.

The perception of stress elicits a varied response that may involve a wide range of behaviors (e.g., escape or avoidance behavior); biological responses; and, in humans, subjective awareness of a distressed emotional state. Stress-related biological responses include psychophysiological reactions, such as changes in skin conductance (e.g., from sweating), muscle tension, and cardiovascular responding (e.g., changes in heart rate), as well as changes in the activation of various brain regions. Alcohol consumption can reduce the magnitude of an organism’s response to stress. This reduction is called stress-response dampening (SRD) (Levenson et al. 1980).

Researchers can measure alcohol’s SRD effects in various ways. Among the most common measures are scales on which respondents are asked to rate their levels of certain emotional states, such as anxiety, tension, nervousness, or apprehension. Another frequently used approach for determining alcohol’s SRD effects involves monitoring physiological responses, most commonly changes in heart rate. Finally, SRD studies sometimes include behavioral measures, such as measures of activity (e.g., the time needed to escape an unpleasant stimulus) and expressive behavior (e.g., facial expressions of negative emotional states).

## Alcohol’s Effects on Stress Responding

By the 1980s researchers had conducted numerous studies to determine whether drinking reduced stress. To the surprise of many investigators, the relationship between alcohol and stress was inconsistent. Alcohol consumption reduced stress in some studies, did not affect stress responses in other analyses, and exacerbated stress in still other investigations (Sayette 1993*a*). (Steele and Josephs [1988] described the latter outcome as the “crying-in-your-beer effect.” ) These contradictory findings led some researchers to conclude that the tension-reduction hypothesis had not been confirmed. Other scientists argued, however, that despite some discrepancies, the study results generally supported the tension-reduction model. Perhaps the most common conclusion was that alcohol’s effects on stress were complex and that further research was needed to specify the conditions under which drinking would most likely reduce stress (see Sayette 1993*a*).

In recent years many studies have been conducted to clarify the relationship between drinking and stress reduction. Two general areas of inquiry emphasized in those analyses assess the personal or individual differences and the situational factors that mediate alcohol’s SRD effects (Wilson 1988). Research on individual differences seeks to identify those people in whom alcohol is most likely to reduce stress. Research on situational factors attempts to determine the circumstances under which alcohol consumption is most effective in reducing stress. The following sections review various individual and situational variables and the roles that they may play in alcohol’s SRD effects.

### Individual Differences

Researchers have suggested that several personal characteristics may influence the extent to which a person is sensitive to alcohol’s SRD effects. These characteristics include a family history of alcoholism, personality traits, extent of self-consciousness, level of cognitive functioning, and gender.

#### Family History of Alcoholism

Children of alcoholics are at heightened risk of becoming problem drinkers compared with children of nonalcoholics (Sher 1991). Scientists are investigating the mechanisms underlying this increased risk. One line of research in this field has examined whether alcohol consumption may produce an enhanced SRD effect and, consequently, provide greater reinforcement^2^ in people at increased risk for alcoholism. These studies have compared the SRD responses of participants with a family history of alcoholism (i.e., family-history positive [FHP] individuals) to the SRD responses of participants without such a family history (i.e., family-history negative [FHN] individuals).

To date, the findings of those investigations have been equivocal. In the first large study conducted in this area, the investigators found that compared with the FHN participants, the FHP participants exhibited increased SRD responses to alcohol on two of five psychophysiological measures tested (Levenson et al. 1987). Conversely, in a subsequent study, a family history of alcoholism did not influence the SRD effect of alcohol (Sayette et al. 1994). Still other studies have suggested that only participants with a multigenerational family history of alcoholism demonstrate an enhanced SRD response to alcohol. This observation indicates that the effects of paternal alcoholism on the SRD response of the offspring can best be assessed in subjects with an extensive family history of alcoholism affecting several generations (e.g., father and paternal grandfather) (Finn et al. 1990).

Several reasons may contribute to the discrepant findings and the difficulties in determining the exact relationship between SRD and family history of alcoholism. Differences in that relationship between FHP and FHN participants may appear smaller than they actually are, because the participants’ classification as either FHP or FHN typically is based solely on self-reports. Although studies demonstrate that such self-reports are generally accurate, an improved assessment of parental alcoholism (e.g., through corroboration by a parent) might strengthen the association between a family history of alcoholism and the relationship between alcohol and stress.

A second confounding factor is that alcohol administration studies only include participants who are of drinking age (i.e., at least 21 years old) and who have not yet developed a drinking problem. Accordingly, many of the FHP individuals at greatest risk for developing alcoholism may be ineligible for study participation because they have already developed a pathological drinking pattern before age 21. Such a selection bias may underestimate the effect of a family history of alcoholism on the impact of alcohol’s SRD effect.

Although some evidence suggests, as discussed in this section, that a family history of alcoholism influences a person’s SRD response to alcohol, many questions remain. For example, researchers are just beginning to identify mechanisms that may underlie the potential relationship between family history and SRD response. In one line of research, investigators are analyzing whether alcohol’s SRD effects may be more pronounced in FHP subjects when blood alcohol concentrations (BACs) are rising (i.e., on the rising limb of the BAC curve) than when BACs are falling (i.e., on the falling limb of the BAC curve) (Sayette 1993*a*). This finding is potentially relevant, because alcohol’s reinforcing effects are thought to be stronger on the rising limb than on the falling limb of the BAL curve (Sher 1991). These investigations are based on evidence that when sober, FHP subjects may exhibit stronger physiological reactions to a variety of stimuli than do their FHN counterparts (see Sher 1991). For example, Finn and colleagues (1990) have hypothesized that children of alcoholics exhibit greater responses to various types of events, regardless of whether those events are stressful (e.g., exposure to an aversive electric shock) or not (e.g., exposure to nonaversive tones, such as a tone with a frequency of 1 kHz and a volume of 70 decibel). Furthermore, some studies have suggested that FHP drinkers are more physiologically reactive to alcohol consumption itself and that this reactivity may affect their subsequent response to a stressor (see Sayette 1993*b*).

#### Personality Traits

Since the early 1980s, researchers have associated certain personality traits that are considered indicative of an elevated risk of alcoholism with an enhanced SRD response to alcohol. For example, Sher and Levenson (1982) found that people who experienced difficulty in controlling their behavior (i.e., who scored high on measures of behavioral undercontrol) experienced increased SRD effects. This and other observations suggest that people with such personality characteristics might be more susceptible to alcohol’s reinforcing effects, including SRD. This increased susceptibility, in turn, could facilitate the development of alcoholism (see Sher 1991).

#### Extent of Self-Consciousness

Hull (1987) proposed that people who are highly self-conscious are most likely to experience alcohol’s SRD effects. According to this self-awareness model, self-conscious people constantly evaluate their own performance and may experience stress if the result of that self-evaluation is negative. Alcohol consumption impairs the drinker’s ability to encode information from the environment with respect to its relevance to the self. Consequently, both the drinker’s self-awareness and the associated stress decline. The stress reduction has a reinforcing effect, thereby increasing the probability of further drinking. Some studies have supported this hypothesis by demonstrating that highly self-conscious people are more sensitive to alcohol’s SRD effects (see Hull 1987; Sayette 1993*a*). Other studies, however, have produced conflicting results.

#### Cognitive Functioning

A person’s cognitive functioning also may influence the extent of his or her SRD response to alcohol. Alcohol has been shown to disrupt the processing of new information in the brain (i.e., cognitive processing). Consequently, alcohol may be particularly disruptive to people with cognitive deficits. To assess the relationship between cognitive functioning and alcohol’s SRD effects, Peterson and colleagues (1992) administered a battery of neuropsychological tests to a group of drinkers to assess their cognitive functioning. The study participants then received a drink before being exposed to a laboratory stressor (i.e., a series of mild electric shocks). The researchers found that the participants with the lowest cognitive performance (i.e., with the greatest difficulty organizing new information) exhibited the greatest SRD response to alcohol. Other investigators also found a similar relationship between cognitive performance, alcohol consumption, and SRD response in a study using a test of “minimal brain dysfunction” (Sher and Walitzer 1986).

#### Gender

Most studies conducted in the alcohol field, including those that have analyzed the relationship between alcohol consumption and stress, have involved only male participants. Only recently have studies been conducted that have included both male and female participants. Early studies of alcohol’s effects on stress that involved both genders indicated that alcohol’s SRD effect differed between men and women (Abrams and Wilson 1979). Subsequently, however, the findings of the largest studies that included participants of both genders suggested that alcohol’s effects on stress seem to be comparable for men and women (see Sayette et al. 1994). Thus, even in studies in which women appeared to be more responsive than men to a stressor when the participants were sober, alcohol consumption did not alter this gender difference (i.e., the women were still more responsive to the stressor), suggesting that the extent of alcohol’s SRD effects was similar in both men and women. It is possible, however, that the presence or absence of gender differences depends on the measures used to determine the stress response. In the studies that detected no gender differences, researchers assessed the stress response using only physiological (e.g., heart rate) and self-report measures. Consequently, other types of measures of emotional response (e.g., analysis of facial expressions associated with emotions) might reveal gender differences in the SRD response to alcohol.

### Situational Factors

Although numerous influences specific to each drinker affect the extent to which he or she experiences alcohol’s SRD effects, the characteristics of the situation in which drinking occurs also modify the drinker’s response to alcohol. Thus, the same person experiences alcohol’s effects differently when drinking at a party with friends than when consuming a drink alone at a bar after a stressful day at work. Two such situational factors that have been shown to affect alcohol’s SRD effects are distraction and the timing of drinking and stress.

#### Distraction

In an attempt to determine the reasons underlying alcohol’s variable effect on stress, Steele and Josephs (1988) proposed that alcohol reduces stress only when drinking occurs in the presence of stimuli that distract the drinker from his or her distress. According to this attention-allocation model, alcohol impairs cognitive processing. Consequently, the drinker can perceive and focus on only the most immediate cues and a situation’s most relevant (i.e., salient) features. Accordingly, the concurrent activity in which a person engages while consuming alcohol helps determine alcohol’s effects. For example, according to the attention-allocation model, drinking in a stressful situation (e.g., after a bad day at work) in the presence of a concurrent pleasant distraction (e.g., at a party with friends) leads to an SRD response, because the drinker perceives only the pleasantly distracting aspect of the situation and cannot focus on the stressor. Conversely, drinking without a concurrent neutral or pleasantly distracting activity (e.g., alone in a bar) does not produce an SRD effect and may even increase stress, because the drinker’s attention focuses on the then-salient stressor.

Several studies have confirmed the hypothesis of the attention-allocation model. In those studies, alcohol consistently induced an SRD response when drinking occurred in combination with a pleasant distraction during a stressful laboratory task. Without such a distraction, however, drinking no longer reduced—and sometimes even intensified—stress^3^ (Curtin et al. 1998; Steele and Josephs 1988). Because most people drink in situations that include distractions, the attention-allocation model suggests that alcohol often will produce SRD effects. Some laboratory studies, however, have produced conflicting results, demonstrating an alcohol-induced stress reduction even in the absence of a distraction (Sayette 1993*a*). Nevertheless, the attention-allocation model provides a plausible explanation for both the stress-reducing and stress-enhancing effects of drinking.

#### Timing of Drinking and Stress

A second situational factor that may affect alcohol’s SRD effects is the time when drinking occurs relative to the stressful experience. Studies have demonstrated that alcohol’s SRD effect will more likely occur when a person consumes alcohol before learning of a stressor rather than after learning of a stressor (Sayette and Wilson 1991). To explain these observations, Sayette (1993*a*) proposed the appraisal-disruption model. According to that model, intoxication impairs the cognitive processes associated with the appraisal of new information. Specifically, drinking may interfere with the initial perception of stressful information by preventing the activation of associated stressful memories and concepts.

The appraisal-disruption model postulates that when intoxication precedes exposure to a stressor, impaired appraisal may reduce stress by protecting the drinker from fully experiencing a stressor. If the stressor has already been appraised sufficiently to cause stress, however, subsequent drinking may no longer reduce that stress. This hypothesis can be illustrated by the following example. Imagine a person who has been invited to a dance but who is not a good dancer and feels highly uncomfortable when having to participate in such an event. If that person consumes alcohol before attending the dance, his or her processing of the stressful information (e.g., a dance partner laughing at him or her) may be reduced. As a result, the person may experience less stress at the dance. If that person consumes alcohol only after arriving at the dance, however, he or she will already have processed the stressful information sufficiently to induce a stress response. Accordingly, subsequent alcohol consumption may not reduce the stress response (unless, of course, the drinker is sufficiently distracted by his or her friends and other events at the dance to “forget” his or her own discomfort, as posited by the attention-allocation model).

A review of more than 30 studies conducted in numerous laboratories provides support for the appraisal-disruption model (Sayette 1993*a*). Among the studies, those in which researchers provided their subjects with alcohol before informing them of an upcoming stressor consistently found that alcohol reduced the participants’ stress. In contrast, alcohol’s effects on stress were extremely variable (i.e., alcohol increased, decreased, or had no effect on stress) in studies in which the investigators informed participants about the stressor before providing alcohol.

The appraisal-disruption model accommodates many of the apparently contradictory findings reported in past investigations. Specifically, the model offers an explanation for why only some experiments detect an SRD effect of alcohol. Nevertheless, several features of the model require further examination. For example, with few exceptions (e.g., Josephs and Steele 1990), studies have not included measures of both the stress response and of cognitive disruption. Consequently, measures of the precise mechanisms posited to underlie alcohol’s disruption of appraisal (e.g., measures of how alcohol affects the activation of stressful memories by a current stressor) should be included in future studies (see Sayette 1993*a*). Furthermore, the appraisal-disruption model does not settle the question of which types of information are most sensitive to alcohol’s effects. For example, researchers still need to investigate whether alcohol selectively disrupts the processing of stressful information (Curtin et al. 1998; Sayette 1993*a*).

## Conclusions

Studies of the relationship between alcohol and stress suggest that drinking can reduce stress in certain people and under certain circumstances. Studies conducted over the past two decades have identified several factors that render certain people particularly susceptible to alcohol’s SRD effects. For example, a family history of alcoholism may increase a person’s likelihood of experiencing those effects. However, some of those studies require further replication and clarification of the mechanisms underlying this enhanced susceptibility. In addition to FHP individuals, alcohol may be effective in reducing stress in people who have difficulty controlling their behavior, are highly self-conscious, or have difficulty organizing new information while sober. Future studies are needed to confirm those relationships.

As researchers identify additional individual factors that influence a person’s SRD response, models will need to be developed that integrate the different variables. For example, Peterson and colleagues (1992) found that participants’ scores on some of their neuropsychological tests were associated not only with the SRD response but also with a family history of alcoholism. These observations suggest that a link may exist between family history, cognitive performance, and the susceptibility to alcohol’s SRD effects.

Scientists also have identified situational variables that modify alcohol’s SRD effects. For example, alcohol has been shown to reduce stress reliably when drinking occurs in the presence of pleasant distractions. Furthermore, laboratory studies suggest that drinking before experiencing a stressor attenuates stress, whereas drinking after experiencing a stressor may have no effect or may even exacerbate stress. These findings, however, require replication in more natural settings outside the laboratory.

Research also is needed to improve understanding of the mechanisms underlying alcohol-induced exacerbation of stress (Curtin et al. 1998; Sayette 1993*a*). For example, scientists must examine the effects of drinking on coping processes during stressful situations. Moreover, studies should investigate whether certain types of information are more resistant than other types to alcohol-related impairment. For example, drinking may differentially affect the processing of positive and negative information, with negative information becoming less accessible than positive information during intoxication (Sayette 1993*a*). Research testing the responses of people exposed to both stressful and positive information should help scientists to better understand the mechanisms underlying alcohol’s ability to reduce stress. Although the evidence for a direct stress-reducing effect of alcohol remains somewhat controversial, researchers have proposed several mechanisms that could underlie alcohol’s SRD effects. These explanations emphasize alcohol’s effect on both the peripheral and central nervous systems.^4^ One study using numerous cardiovascular measures found a response pattern suggesting that the SRD response may be restricted to those cardiovascular functions that are regulated by a certain subset of peripheral nerves (i.e., beta-adrenergic nerves) (Levenson et al. 1987). Other studies, however, have not confirmed those findings (see Sayette 1993*a*). Furthermore, data from a variety of sources have led to the alternative hypothesis that alcohol’s SRD effects result from alcohol-induced changes in central nervous system activity (Koob and Bloom 1988; Sayette 1993*a*; Sher 1987). To date, the precise pharmacological mechanisms underlying alcohol’s SRD effects remain unclear (Koob and Bloom 1988).

## Footnotes

1 *Winter Scene *by Alcaeus of Mytilene, quoted in Lattimore 1960, p. 44.

2 Reinforcement occurs when a person experiences alcohol effects that motivate continued drinking.

3 In those studies, stress was intensified in the presence of a mental-distraction task.

4 The central nervous system includes the brain and the spinal cord. The term “peripheral nervous system” refers to all motor and sensory nerves outside the central nervous system.
